# Comparative Response of Platelet fV and Plasma fV to Activated Protein C and Relevance to a Model of Acute Traumatic Coagulopathy

**DOI:** 10.1371/journal.pone.0099181

**Published:** 2014-06-12

**Authors:** James E. Campbell, Michael Adam Meledeo, Andrew P. Cap

**Affiliations:** United States Army Institute of Surgical Research, Coagulation and Blood Research Program, JBSA-FT Sam Houston, Houston, Texas, United States of America; Institut National de la Santé et de la Recherche Médicale, France

## Abstract

**Background:**

Acute traumatic coagulopathy (ATC) has been linked to an increase in activated protein C (aPC) from 40 pM in healthy individuals to 175 pM. aPC exerts its activity primarily through cleavage of active coagulation factor Va (fVa). Platelets reportedly possess fVa which is more resistant to aPC cleavage than plasma fVa; this work examines the hypothesis that normal platelets are sufficient to maintain coagulation in the presence of elevated aPC.

**Methods:**

Coagulation responses of normal plasma, fV deficient plasma (fVdp), and isolated normal platelets in fVdp were conducted: prothrombin (PT) tests, turbidimetry, and thromboelastography (TEG), including the dose response of aPC on the samples.

**Results:**

PT and turbidimetric assays demonstrate that normal plasma is resistant to aPC at doses much higher than those found in ATC. Additionally, an average physiological number of washed normal platelets (200,000 platelets/mm^3^) was sufficient to eliminate the anti-coagulant effects of aPC up to 10 nM, nearly two orders of magnitude above the ATC concentration and even the steady-state pharmacological concentration of human recombinant aPC, as measured by TEG. aPC also demonstrated no significant effect on clot lysis in normal plasma samples with or without platelets.

**Conclusions:**

Although platelet fVa shows slightly superior resistance to aPC's effects compared to plasma fVa in static models, neither fVa is sufficiently cleaved in simulations of ATC or pharmacologically-delivered aPC to diminish coagulation parameters. aPC is likely a correlative indicator of ATC or may play a cooperative role with other activity altering products generated in ATC.

## Background

Uncontrolled hemorrhage remains the leading cause of preventable death following trauma [1], [2]. Severe trauma featuring both tissue damage and shock is associated with abnormal results in *ex vivo* coagulation function tests, independent of iatrogenic perturbations caused by hemodilution with crystalloid or colloid [3], [4]. This early coagulopathy has been linked with increased transfusion requirements, mortality, and morbidity [5], [6]. Termed acute traumatic coagulopathy (ATC), this syndrome is defined by an international normalized ratio (INR) greater than 1.2 and occurs within the first thirty minutes following trauma [3], [4], [7], [8]. ATC is associated with a decrease in total protein C (PC) and an increase in activated protein C (aPC). It has been hypothesized that ATC is caused, at least in part, by the proteolytic functions of aPC [9], [10].

PC is activated in the presence of α-thrombin (fIIa), a process which is greatly accelerated when fIIa is complexed with thrombomodulin (TM) and endothelial protein C receptor (EPCR) [11]. aPC is a serine protease that exerts anticoagulant effects primarily through the inactivation of the co-enzyme factor (f) Va. aPC also inactivates fVIIIa but has a much higher affinity for fVa; at least one study demonstrates that the thrombin-inhibiting ability of aPC is primarily through its actions on fVa [12]. Notably, depletion of both fV and fVIII is reported in ATC [10]. aPC inactivates fVa primarily through initial cleavage at Arg506 and subsequent cleavage at Arg306, accelerating further cleavage and preventing assembly of the prothrombinase complex, completing a negative feedback loop for fIIa and subsequent PC activation [12]. This process significantly decreases rates of and maximum thrombin generation in closed systems with soluble TM [12] and in TM-expressing endothelial cells [13], [14]. In normal hemostasis, the presence of endothelial-expressed TM aids in restricting a clot to dimensions that are congruent with the vascular wound area [15], presumably through the activation of PC. Whether this process functions normally in severe trauma with damage to large microvascular beds remains to be determined.

High concentrations of aPC are thought to facilitate fibrinolysis, possibly through binding plasminogen activator inhibitor-1 (PAI-1) [16], [17]. This is further corroborated by a correlation between decreasing protein C and PAI-1 with increasing tissue plasminogen activator (tPA) and d-dimer [8], [18]. However, the profibrinolytic properties of aPC are dependent on its downstream effects on thrombin-activatable fibrinolysis inhibitor (TAFI); inactivation of fVa reduces fIIa production which reduces TAFI activation [19]. In trauma, activated platelets release Platelet Factor 4 (PF4), which acts as a soluble cofactor promoting aPC generation by the fIIa-TM complex while simultaneously inhibiting the activation of TAFI by fIIa-TM [20]. This complexity of mechanisms suggests that the interaction of aPC and PAI-1 may actually be insignificant since the reduction in fIIa production by aPC could lead to a delay in fibrinolysis [21].

Since ATC is characterized by PC activation, depletion of fV/fVIII, and possible fibrinolytic effects from aPC neutralization of PAI-1, it is posited that a large-scale activation of PC in massive trauma is directly responsible for the poor outcomes associated with ATC. Circulating aPC levels have been estimated at about 40 pM in healthy individuals [22]. It has been suggested that ATC is induced by aPC levels near 10 ng/mL (∼175 pM), reflecting activation of approximately 0.2% of the total average circulating protein C (3750 ng/mL; ∼65.8 nM) [10]. Drotrecogin alfa (activated) (DrotAA) is recombinant human aPC previously marketed for severe sepsis under the brand name Xigris (Eli Lilly, Indianapolis, IN); the median steady-state concentration of DrotAA following an infusion of 12–30 µg/kg/hr is 45 ng/mL (∼790 pM), dissipating below 10 ng/mL within two hours [23]. Both 175 pM and 790 pM are considered anti-thrombotic within their clinical contexts, sufficient to reduce plasma fV levels [10], [24], [25].

fV exists in two pools distinguished by differences in structure, function, and susceptibility to inactivation: the platelet fV fraction (∼20%) has been established as being refractory to aPC cleavage compared to the plasma fraction (∼80%) [26]–[28].

Previous studies have shown that hemostatic thrombin generation can be produced by introducing platelets in patients suffering from fV-deficiency or in plasma treated to be fV-deficient [29]. We hypothesized that a physiologically normal concentration of healthy platelets would possess fV capable of resisting the anti-coagulant effects of aPC at the reported ATC-inducing concentration. To test this hypothesis, we investigated the concentration of aPC required to induce significant delays in clot time, decrease clot strength, or induce fibrinolysis as described in ATC, as well as the number of platelets necessary to correct aPC-perturbed coagulation parameters. The results indicate that the concentration of aPC required to induce an acute coagulopathy in an otherwise healthy patient's plasma is well beyond reported pathophysiologic levels. In platelets especially, fV is resistant to moderately high aPC in clinically relevant static or low-shear models such as thromboelastography.

## Methods

### Ethics Statement

This study was conducted under a protocol reviewed and approved by the San Antonio Military Medical Center Institutional Review Board, and in accordance with good clinical practices. Written informed consent was obtained from blood donors.

### Blood and Plasma Preparation

De-identified fresh whole blood from healthy donors (50 mL) was collected in 4.5 mL BD Vacutainer tubes (10.9 mM buffered sodium citrate; Becton-Dickinson, Franklin Lakes, NJ); each sample was collected from a new donor and processed immediately.

Plasma samples were prepared through a series of centrifugation steps: 200-g for 10 min with minimal braking separated platelet-rich plasma (PRP) which was collected and stored at 22 °C; additional centrifugation of the sample at 2,000-g for 10 min isolated platelet-poor plasma (PPP). PPP was additionally centrifuged at 10,000-g for 4 min to generate platelet-free plasma (PFP) and kept on ice until use.

Frozen citrated fV immunodepleted plasma (fVdp; Haematologic Technologies, Essex Junction, VT) was rapidly thawed in a 37 °C water bath immediately prior to use. Per the manufacturer, this lot of plasma (BB0926) has a prothrombin time (PT) of 53.7 s, activated partial thromboplastin time (APTT) of 110.9 s, fV<1% normal, and other coagulation factors within normal ranges. Frozen citrated fVIII chemically depleted plasma (fVIIIdp; Haematologic Technologies) was handled in the same manner; this lot of plasma (DD0220B) has a PT of 12.1 s, an APTT of 91.8 s, fVIII <1% normal, and other coagulation factors in their normal ranges.

Corn trypsin inhibitor (CTI; 20 µg/mL; Haematologic Technologies) was added to all samples to assure contact pathway blockade for 1 hour. The lipidated recombinant tissue factor Innovin (Dade-Behring, Marburg, Germany) was used to initiate clotting at a 1∶5000 dilution. Citrated plasma was recalcified immediately prior to experiments with 15 mM CaCl_2_ (Sigma-Aldrich, St. Louis, MO).

### PT/INR Analysis

The STA-R Evolution (Diagnostica-Stago, Parsippany, NJ) was used to collect PT data. Briefly, 100 µl STA Neoplastine CI Plus 10 reagent (freeze-dried thromboplastin from rabbit containing calcium) was prepared according to manufacturer's instructions and mixed with 50 µl plasma (pre-incubated at 37 °C). The Mean Normal PT is listed as 13.5 s for this device, and INR is calculated by the formula INR  =  (Sample PT/Mean Normal PT)^ISI^, where ISI is the International Sensitivity Index, given as 1.21 for these reagents.

### Turbidimetric Assays

The Spectramax M5e plate reading spectrophotometer (Molecular Devices, Sunnyvale, CA) was used to collect turbidimetric absorbance measurements which are correlated with fibrin network formation. The spectrophotometer was run in kinetic absorbance mode (405 nm) for 90 min with 20 s intervals at 37 °C. Total volume per well was 100 µL. Assays were conducted in 96-well flat clear bottom polystyrene non-treated microplates (Costar 3615, Corning, Tewksbury, MA). Results were exported from SoftMax Pro 5.4.5 (Molecular Devices) for analysis.

The effects of purified aPC (Lot CC0118, Haematologic Technologies) on clot time in plasma were examined by performing a serial dilution of aPC concentrations from 0.001–100 nM, with 100 nM being ∼50% above the theoretical average maximum amount of aPC that can exist systemically (∼65.8 nM). This protein was used in all aPC-inclusive assays and has been demonstrated to have full physiologic function [30]–[34].

aPC concentrations were incubated with plasma for 3 min at room temperature. Innovin was added to plasma immediately before distribution to the 96-well plate (preloaded with CaCl_2_). The ability of recombinant human soluble EPCR (Novoprotein, Summit, NJ) and phosopholipid in the form of an emulsion of phosphatidyl choline (PC), phosphatidyl serine (PS), and sphingomyelin (SM) at a 42∶28∶30 molar ratio (Rossix, Mölndal, Sweden) to modulate aPC anticoagulant activity were also evaluated via the turbidimetric assay.

### Isolated Platelets

Platelets were washed and isolated from PRP similarly to the procedure of Mustard et al [35]. Briefly, 0.02 U/mL apyrase (Sigma-Aldrich) and 1.0 µM prostaglandin I2 (PGI_2_; MP Biomedicals, Solon, OH) were added to PRP to prevent platelet activation. PRP was centrifuged at 1,000-g for 10 min at room temperature without braking. Following plasma aspiration, the platelet pellet was gently resuspended with 10 mL of modified Tyrode's albumin buffer (MTAB: 137 mM NaCl, 2.68 mM KCl, 11.9 mM NaHCO_3_, 0.43 mM NaH_2_PO_4_, 1.0 mM MgCl_2_, 5 mM HEPES, 0.35% BSA, 5.55 mM D(+)Glucose). Additional apyrase (0.01 U/mL) and PGI_2_ (0.5 µM) were added; the platelets were centrifuged again at 1,000-g for 10 min at room temperature without braking, and the pellet was resuspended at high concentration in 300 µL fVdp. Platelets were counted using a Coulter Ac-T diff2 hematology analyzer (Beckman Coulter, Inc., Indianapolis, IN) and adjusted to the desired concentration by the addition of fVdp prior to use.

### Thromboelastography

Thromboelastographic (TEG) assays were conducted with the Haemoscope TEG 5000 Coagulation Analyzer (Haemoscope Corp., Niles, IL). The clinical availability and common usage of this device provides an alternative measurement of blood clotting to the turbidimetric assay, particularly in samples containing red blood cells which interfere with the opacity.

The effects of aPC dose were determined on fVdp and normal plasma samples with or without washed platelets, using Innovin and CaCl_2_ to activate coagulation. The total volume per sample was 300 µL; samples were run for 3 h.

### fVIII activity

The dose-response of fVIII activity to aPC was evaluated using the STA - Deficient VIII assay on the STA-R Evolution per manufacturer's instructions.

### Statistics

For all experiments, n≥3. Variance component estimates (JMP software, SAS Institute, Cary, NC) were used to demonstrate that sample-to-sample random effects comprised an insignificant portion of the observed variance. Prism 5 (GraphPad Software, La Jolla, CA) was used to generate graphs and perform two-way analysis of variance with repeated measures and pair-wise comparison Bonferroni posttests.

## Results

### Nanomolar aPC Is Required to Prolong PT/INR

**Figure 1:**
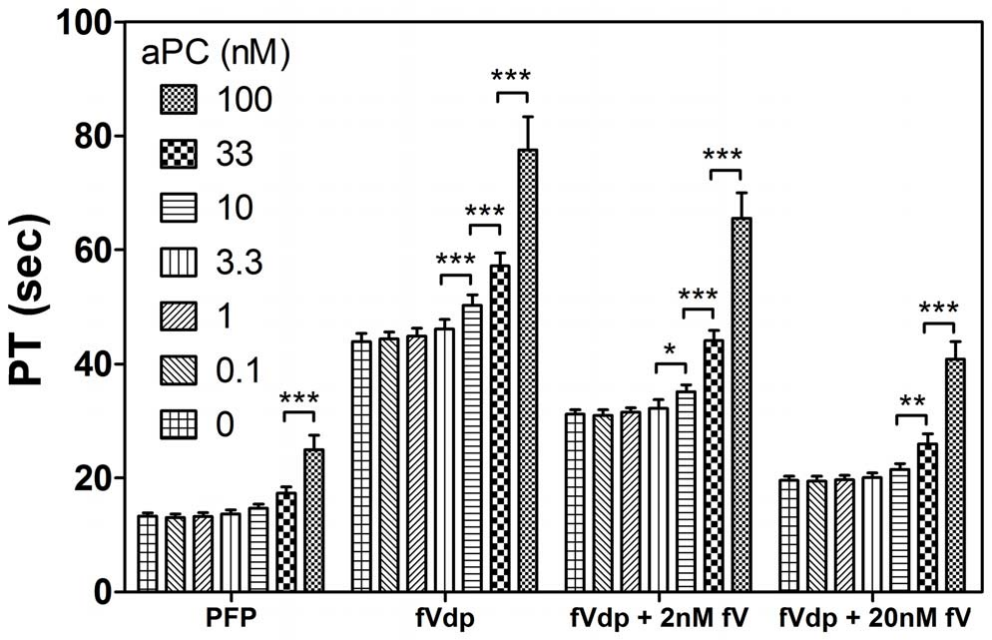
Prothrombin times of plasmas treated with aPC. Increasing aPC from 0 to 3.3 nM has no effect on PT regardless of fV levels. In fVdp and fVdp supplemented with 2(***: p<0.001 for the null hypothesis of the indicated samples; **: p<0.01; *: p<0.05). Means of three samples are shown with standard deviation.

PT measurements in fVdp are delayed compared to normal PFP (43.9 s in fVdp control versus 13.3 s in PFP control, Figure 1). The addition of a 2 nM concentration of fV protein to fVdp had a slightly restorative effect (PT = 31.2 s), and 20 nM fV reduced PT to 19.6 s in control fVdp samples.

A dose-response analysis of exogenous aPC illustrates that normal and even fV-deficient plasmas are resistant to aPC effects on PT at levels below 10 nM. In normal PFP, there is no statistically significant increase in PT until the aPC concentration exceeds 33 nM. In the fVdp samples, the effects of aPC are noticeable above 3.3 nM. The addition of 2 nM fV slightly reduces the effect of increasing aPC from 3.3 nM to 10 nM, and a 20 nM fV supplementation is resistant to aPC-induced PT prolongation until the concentration of aPC is greater than 10 nM. Calculated INR values ranged from 1.0 (PFP with aPC≤1 nM) to 8.3 (fVdp with 100 nM aPC).

### Nanomolar aPC Is Required to Delay Fibrin Crosslinking

**Figure 2:**
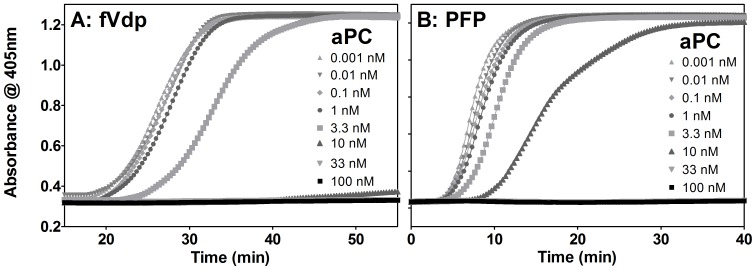
Turbidimetric assay of platelet-free plasmas treated with aPC. The optical density of a plasma sample increases as fibrin crosslinking occurs. (A) fVdp is unaffected by aPC at concentrations of 1 nM and below, while crosslinking is significantly delayed at 3.3 nM and prevented at 10 nM or higher concentrations. (B) In PFP, a slight but insignificant delay is seen at 3.3 nM aPC, and while plasma with 10 nM aPC will clot, the fibrin crosslinking is delayed. Doses of 33 or 100 nM again prevent crosslinking. Note that the time ranges on the x-axis are different as fVdp is inherently delayed in coagulation. Means of three samples are shown.

Optical density of plasma increases with fibrin crosslinking [36]. A turbidimetric assay was used to observe the effects of aPC on fibrin crosslinking following activation with Innovin (Figure 2).

aPC had no significant effect on fVdp fibrin crosslinking (Figure 2A) at a dose of 1 nM or lower (initiation of clotting occurred at approximately 19 min), although as in the PT study, clotting was inherently delayed in fVdp (note x-axis range differences between 2A/2B). In fVdp, 3.3 nM aPC delayed crosslinking to approximately 24 min (p<0.05); 10 nM aPC or higher prevented crosslinking within the 90 minute measurement window (p<0.001).

For PFP (Figure 2B), there was a significant delay in fibrin crosslinking starting at 10 nM aPC (p<0.05).

### Platelet or Plasma-Derived fV Restores Coagulation Parameters in fVdp

**Figure 3:**
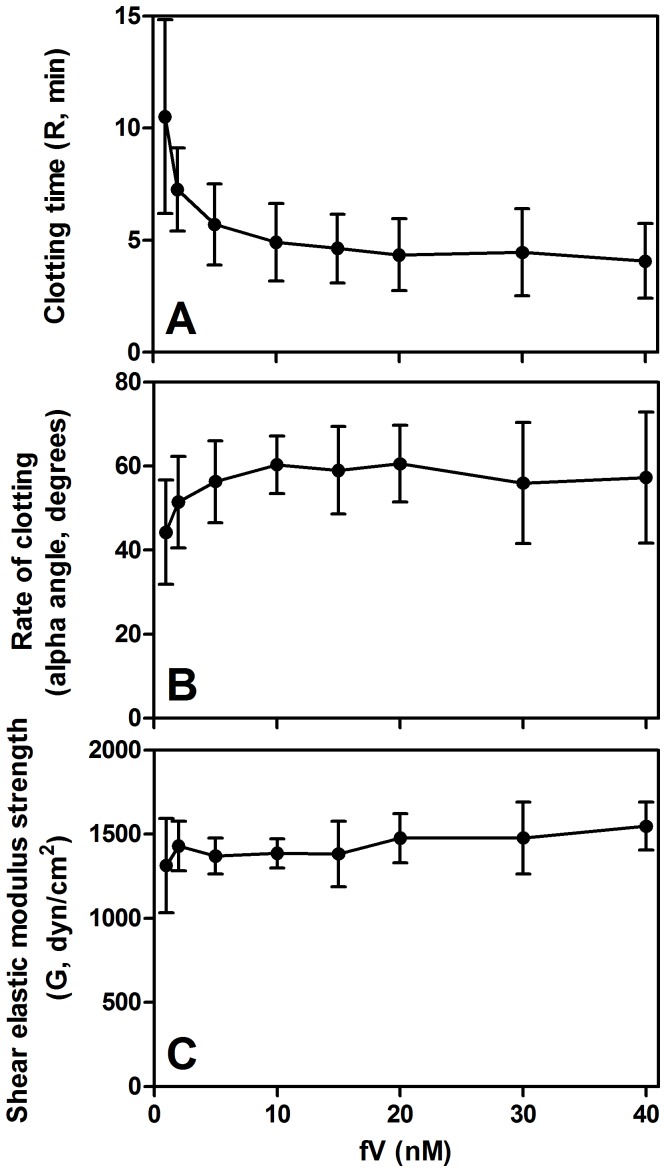
fV protein supplementation effects on TEG coagulation measurements in fVdp. (A) Increasing fV has a profound effect on clotting time at low concentrations (≤10 nM), above which little improvement is observed, reaching a minimum clot time of approximately 4 min. (B) Similarly, the alpha angle increases rapidly as fV increases up to 10 nM, after which the angle is consistently near 60°. (C) Clot strength is unsurprisingly unaffected by increasing the amount of fV present (so long as there is some fV present to form the prothrombinase complex). Means of three samples with standard deviation are shown.

The effect of fV on the coagulation response profile in TEG (Figure 3) was determined by adding fV to fVdp over concentrations from 1–40 nM (average normal is 20 nM) [37]. R-time (clot initiation time, Figure 3A) decreases as fV increases from 1–10 nM and then stabilizes at a minimum time of 4 min. The alpha-angle (clot formation rate, Figure 3B) follows an inverse pattern, increasing as fV increases from 1–10 nM, to a plateau of approximately 60°. The G-value (maximum shear elastic modulus, strength of clot, Figure 3C) is unaffected by fV levels.

**Figure 4:**
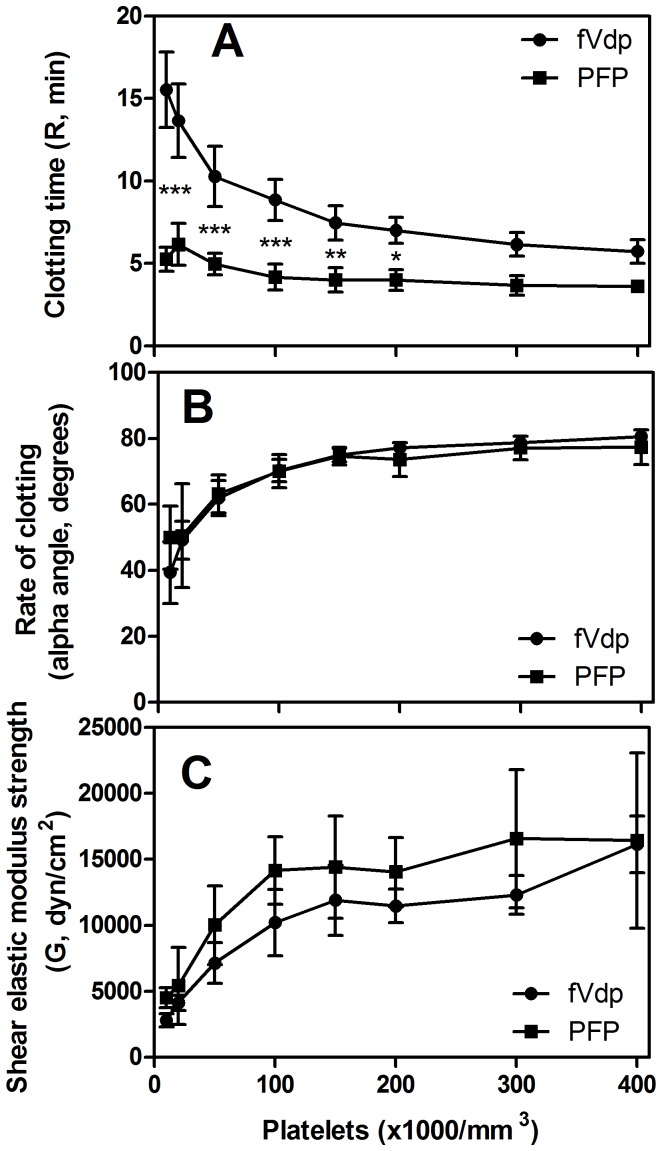
Platelet supplementation effects on TEG coagulation measurements in fVdp and normal PFP. (A) While increasing the number of platelets in PFP has very little effect on the initiation of coagulation, a much more powerful response is observed in fVdp as platelets are increased from 10,000/mm^3^ to 100,000/mm^3^ (***: p<0.001 for the null hypothesis that PFP = fVdp; **: p<0.01; *: p<0.05; n.s.: not significant). Diminishing returns are seen in higher numbers of platelets, with no significant differences observed in the 300,000/mm^3^ and 400,000/mm^3^ concentrations of platelets compared with equivalent numbers in PFP. (B) Increasing platelets from 10,000/mm^3^ to 100,000/mm^3^ dramatically improves the alpha angle; the effect plateaus above 150,000 platelets/mm^3^. No alpha angle differences are observed due to the presence of fV protein in the plasma. (C) Similar to rate of clotting, strength of clot rises rapidly with increasing platelet counts until 100,000/mm^3^ where a plateau occurs. No significant differences are observed between fVdp and normal PFP with equal numbers of platelets. Means of three samples with standard deviation are shown.

Similarly to the direct addition of fV protein, increasing platelet counts from 10,000–400,000 platelets/mm^3^ decreased TEG R-time of fVdp, although increasing above 200,000 platelets/mm^3^ resulted in minimal improvements to clot time, rate of clotting, or clot strength (Figure 4). In normal PFP, clotting time did not change significantly across the range of platelet counts (Figure 4A). Comparing an equivalent number of platelets suspended in either fVdp or normal PFP, significant differences in clot time are seen only at counts ≤200,000 platelets/mm^3^. The alpha-angle (Figure 4B) and G-value (Figure 4C) of equivalent platelet concentrations in fVdp and PFP were statistically indistinguishable.

**Figure 5:**
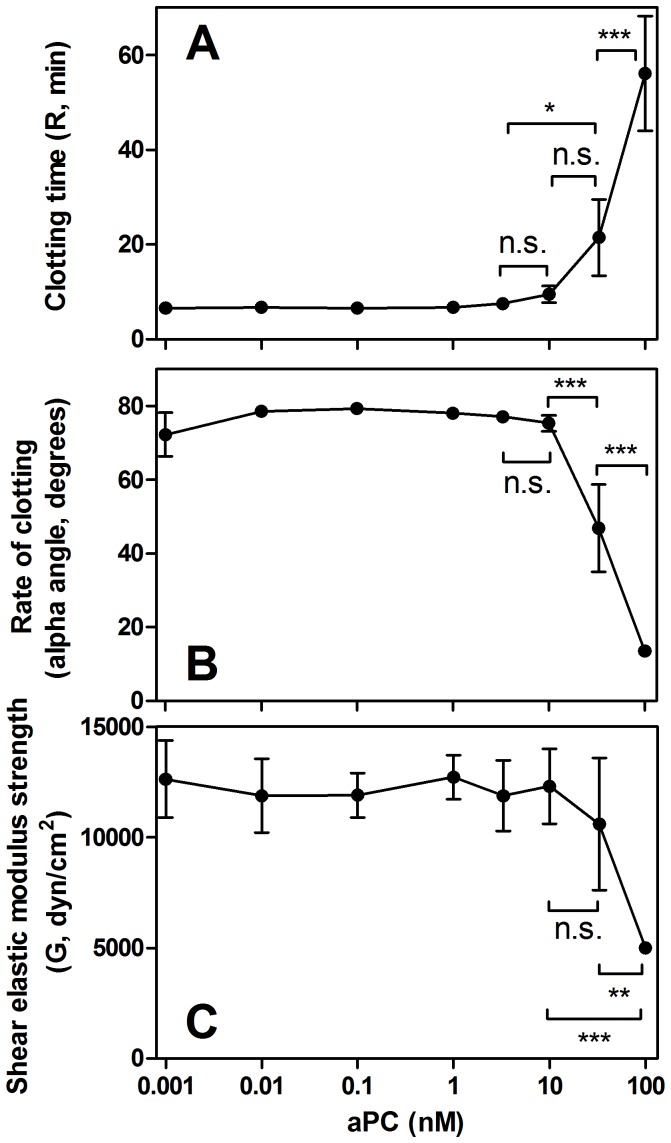
Platelets are resistant to the anti-coagulant effects of aPC as measured in TEG. For fVdp containing 200,000/mm^3^, the (A) R-time (clotting time), (B) alpha angle (rate of clotting), and (C) G-value (clot strength) are only strongly affected by aPC at a concentration of 100 nM compared to all lower doses. The 33 nM dose of aPC demonstrated a significant difference versus all lower concentrations in the alpha angle and all lower concentrations except 10 nM for R-time. G-value was not significantly affected except by the 100 nM aPC dose, although the trend is observable at 33 nM (***: p<0.001 for the null hypothesis that the indicated samples are equal; **: p<0.01; *: p<0.05; n.s.: not significant). Means of three samples with standard deviation are shown.

Washed, non-activated platelets at a concentration of 200,000 platelets/mm^3^ in fVdp eliminated the anti-coagulant effect of aPC up to 10 nM, greatly above systemic concentrations measured in either ATC or DrotAA treatment (Figure 5A). At 33 nM aPC dose, the mean clotting time increased to 21.5 min (p<0.05), and at 100 nM aPC the clot time was extended to 56.1 min (p<0.001).

Significant differences in alpha-angle (Figure 5B) were not observed until 33 nM aPC when it changed from an average 76.8° at 0.001–10 nM aPC to 46.8° at 33 nM aPC and 13.6° at 100 nM aPC (p<0.001 for both compared to lower doses).

Similarly, G-value (Figure 5C) was nearly unaffected by even very high aPC doses. Clot strength was significantly decreased only at 100 nM aPC (p<0.001).

The variance in coagulation parameters found at high aPC concentrations (Figure 5) indicates that individual patients have different thresholds at which they are susceptible to aPC effects, likely due to variability in individual fV availability or other aspects of coagulation factor structure and function.

### aPC and tPA Have No Synergistic Effect on Clot Lysis In Vitro

Activation of PC in ATC may derepress fibrinolysis through aPC degradation of plasminogen activator inhibitor (PAI-1). We examined fibrinolysis in the presence or absence of both aPC and tPA. tPA is released by endothelium and activates plasminogen to plasmin. Plasmin then cleaves crosslinked fibrin, reducing clot size and stability. The pro-fibrinolytic effects of aPC and possible synergy with tPA were evaluated by adding doses corresponding to the proposed ATC level of aPC (175 pM) and the pharmacologic steady-state concentration of DrotAA (750 pM) to normal PFP and PRP (normalized to 200,000 platelets/mm^3^) in combination with doses of tPA known to produce specific fibrinolytic effects (0, 1, 1.5, and 2 nM) [38].

**Figure 6:**
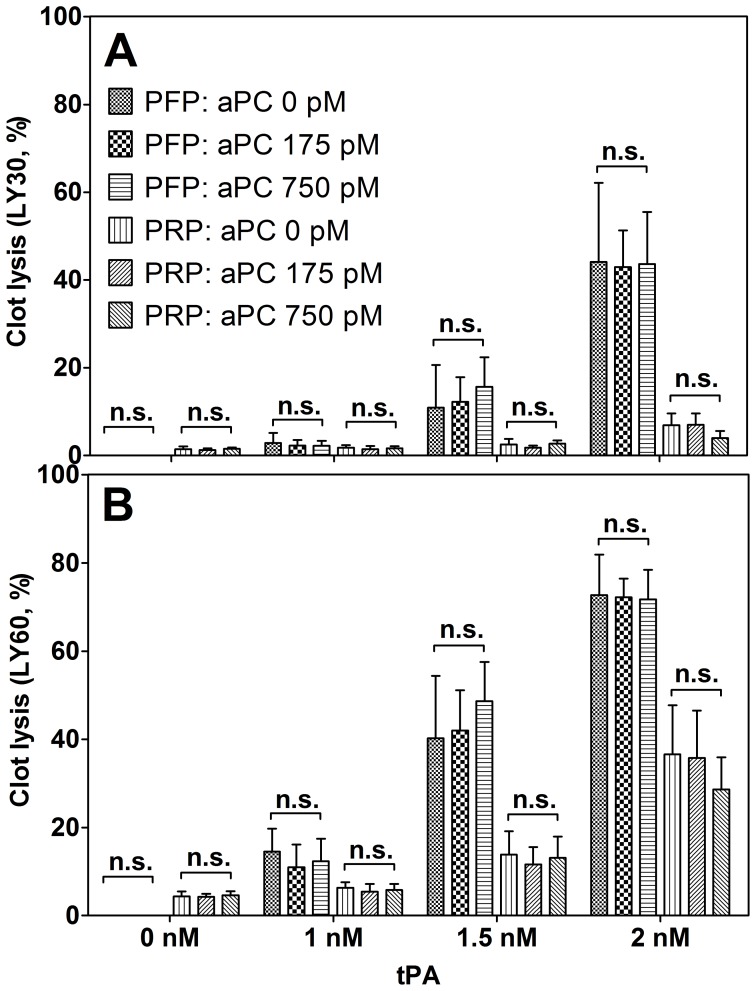
aPC does not induce fibrinolysis inherently in the presence or absence of tPA. Clotting parameters of normal PFP and PRP (normalized to 200,000 platelets/mm^3^) treated exogenously with aPC and tPA were measured in TEG. Clot lysis parameters (A) LY30 and (B) LY60 (percentage of clot lysis at 30 and 60 min, respectively) demonstrate that for a given dose of tPA (0, 1, 1.5, or 2 nM), the dose of aPC (0, 175, or 750 pM) has no effect on clot lysis. The doses of aPC correspond to those found in trauma and the steady-state pharmacological dose. Concentrations of tPA were chosen such that a known response would occur in the absence of aPC. In the absence of tPA, PFP did not experience any lysis while PRP had a limited degree of clot lysis. However, for all non-zero doses of tPA, PFP experienced a greater degree of lysis than PRP. Means of three samples with standard deviation are shown.

Increasing tPA resulted in the expected increase in clot lysis in both plasmas, but increasing aPC had no synergistic effect with tPA; pair-wise comparisons indicated no significant difference in clot lysis between APC concentrations for a given amount of tPA (Figure 6).

### Effects of aPC on Phospholipid Acceleration of Fibrin Formation

Because the phospholipid layer plays an important role in coagulation (of central importance in the activation of fX and thrombin), be it from the platelet, the endothelium, or the microvesicles which exist normally and are elevated in trauma and disease states, it is of some interest to evaluate how the dose-response of phospholipids (PL) modulates the anticoagulant effects of aPC. As an aside, PL is also required for the activation of PC. We examined the effects of PL on coagulation in the presence of aPC by performing a dual dose-response experiment on normal PFP samples. It has previously been shown that thrombin generation rates of platelets within the physiological range correspond to a dose of 1–2 µM PL (a mixture of 25∶75 PS:PC was used in that study) [39].

**Figure 7:**
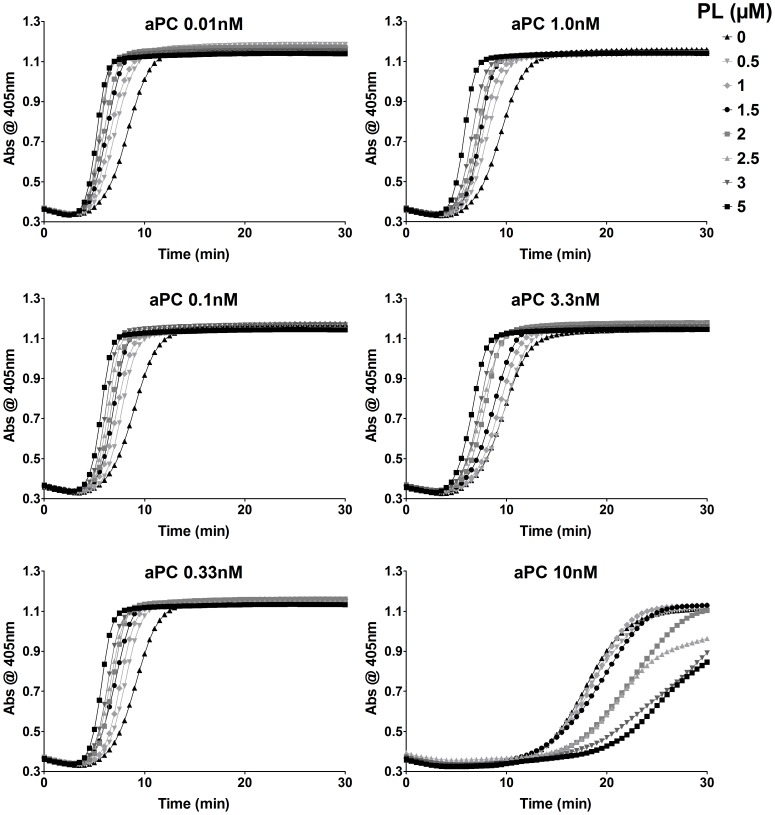
aPC concentrations below 10 nM have no significant effect on PL acceleration of clotting in PFP. In the turbidimetric assay, fibrin crosslinking corresponds to an increase in absorbance at 405

Using the turbidimetric assay, fibrin crosslinking was measured over a ranges of 0–5 µM PL (a mixture of 28∶42∶30 PS:PC:SM) and 0.01–100 nM aPC (Figure 7). As expected, increasing the amount of available PL reduced the time for the initiation of fibrin crosslinking and increased the rate of fibrin crosslinking but had no effect on the maximum absorbance for low concentrations of aPC (which again had negligible effects below 1 nM). At 3.3 nM aPC, a delay in fibrin crosslinking is observed regardless of PL concentration; at 10 and 33 nM concentrations of aPC, PL no longer provides a procoagulant effect. No crosslinking was observed at 100 nM aPC.

### aPC Anticoagulant Activity Is Not Affected by sEPCR Alone

Brief studies were conducted to evaluate the modulation of aPC's anticoagulant activity by EPCR. A commercially available soluble recombinant human EPCR protein was evaluated in the same dual dose response turbidimetric assay (with EPCR instead of PL). Soluble EPCR (sEPCR) exists at a physiological concentration of 2.5 nM in normal individuals and can be elevated as much as five-fold in inflammatory conditions such as systemic lupus erythematosus [40]. However, over the range of 0–200 nM sEPCR, no effect on fibrin crosslinking was observed beyond that generated by aPC alone (data not shown). sEPCR has previously been shown to inhibit the anticoagulant activity of aPC but only in the presence of PL (although not through direct binding to PL vesicles) [41].

### Nanomolar aPC is Required to Degrade fVIII Activity

The degradation of fVIIIa by aPC has a negligible effect on thrombin generation particularly compared to those effects seen through aPC lysis of fVa [12]. For purposes of hemostasis maintenance, fVIII is protected from proteolysis following synthesis through formation of a complex with von Willebrand Factor (VWF), and conversely fVIIIa has other inactivation and clearance mechanisms primarily found in the liver [42], [43].

**Figure 8:**
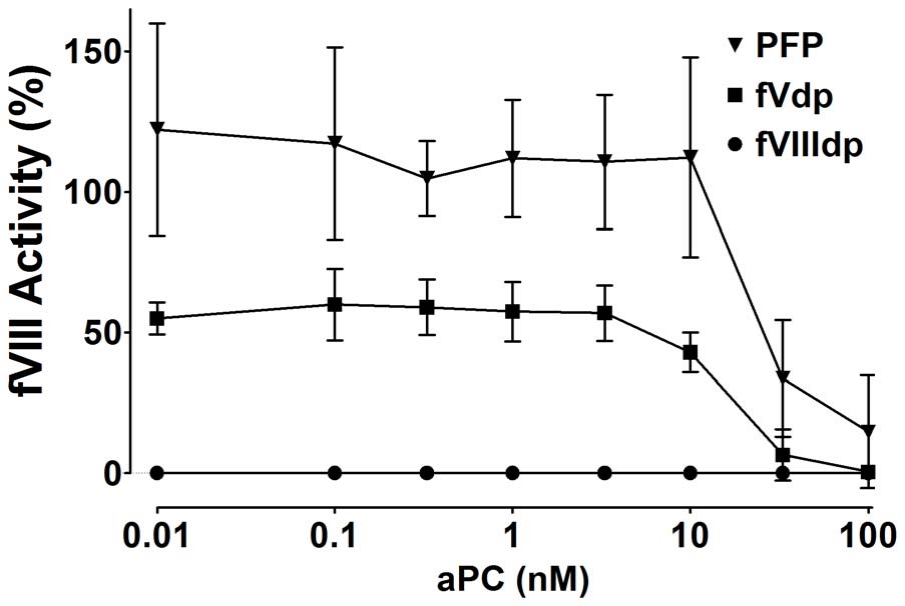
Nanomolar amounts of aPC are required to degrade fVIII activity. In fresh PFP, no change in fVIII activity by aPC was observed until a dose >10 nM (p<0.001). fVdp showed a similar trend (p<0.05 that 3.3 nM and 100 nM aPC are different); fVIIIdp was used as a negative control. The mean and standard deviation of four PFP samples is shown; fVdp and fVIIIdp are means and standard deviations of two technical replicates.

The activity of fVIII was examined for the same aPC dose-response. In fresh PFP, no change in fVIII activity by aPC was observed until a dose >10 nM (p<0.001) (Figure 8). Studies on fVdp showed a similar trend (p<0.05 when comparing 3.3 nM and 100 nM aPC); fVIIIdp was used as a negative control and showed <1% activity throughout.

## Discussion

fVa acts as a cofactor of fXa, increasing the rate of prothrombin activation over 600-fold compared with phospholipid-associated fXa alone [44]. Although fV^-/-^ is embryonic lethal in mice [45], trace amounts of fV found in patients with severe fV-deficiency appear to be sufficient to allow adequate hemostasis [29]. This is partially due to the low level of tissue factor pathway inhibitor in these patients which may act as a compensatory mechanism by improving thrombin generation [46]. However, adequate hemostasis in individuals with normal to low levels of platelet fV and only traces of plasma fV reflects the importance of platelet function and the platelet fV pool. Co-localization of fVa and phospholipid as a result of platelet deposition accelerates thrombin generation and fibrin formation/crosslinking at sites of injury while maintaining flow within vessels [46].

The present results and others [26]–[28] illustrate the relative importance of the platelet fV fraction, although nanomolar concentrations of aPC are required to significantly affect clotting in both normal and fV-deficient plasmas. At 10 ng/mL (175 pM), the concentration of aPC associated with ATC [10], no alterations are observed in PT, clotting time, rate of clot formation, strength of clot, or any other metrics obtainable by either turbidimetry or thromboelastography. As shown in other models, aPC did not induce fibrinolysis except at significantly higher doses than those obtained by administration of DrotAA [47], [48], and no synergy in clot lysis is observed with the addition of aPC to tPA at these relevant concentrations. This may be explained by the complexity of aPC interactions with the fibrinolytic system: on one hand, aPC has been shown to decrease PAI-1 activity [49] (or vice versa [50]); on the other hand, potential reductions in thrombin generation may reduce thrombin-dependent inactivation of PAI-1 [21].

Microvesicles are found in healthy individuals and have been shown to generate small amounts of thrombin which could lead to activation of PC [51], corresponding to the expected homeostatic function of aPC in the healthy individual as a negative feedback regulator of coagulation. In a trauma or sepsis scenario, these microvesicles may have a different function; large amounts of aPC are capable of inducing the formation of microvesicles featuring associated EPCR as shown through *in vitro* cell culture studies (aPC dose of 6.25–100 nM) [52] and in septic patients undergoing DrotAA treatment (standard infusion of 24 µg/kg/hr) [53]. As PC associates with these EPCR-bearing microvesicles it can be activated and perform its anticoagulant proteolytic functions on fVa and fVIIIa. However, the brief studies conducted in this report with the individual components of these aPC-EPCR-microvesicles (using soluble EPCR and a PL emulsion) did not elucidate the mechanisms by which differences in the efficiency of soluble aPC or EPCR-microvesicle bound aPC might be observed. The effects of the combination of sEPCR and PL vesicles were not studied, as it has already been demonstrated that the sEPCR-aPC complex does not bind to PL vesicles [41].

These results confirm and expand those from previous studies examining the anticoagulant effects of aPC in purified systems. Efficacy of aPC at nanomolar concentrations has been shown to be reduced in the presence of platelets but not phospholipid alone [54], and aPC is ineffective at inhibiting both platelet adhesion and fibrin formation in blood flow models except at extreme superphysiological concentrations (16 µg/ml; 285 nM) [55]. aPC has also been shown to be incapable of affecting activation of platelets by thrombin-receptor-agonist-peptide-6 (TRAP) or ADP at DrotAA pharmacological doses (45 ng/ml; 790 pM) and even five-fold higher concentrations [56], while activation of platelets by arachadonic acid, ADP, and collagen was unaltered even at very high aPC doses (10 µg/ml; 178 nM), although that extreme concentration did inhibit activation of platelets by recombinant tissue factor [57]. It has been hypothesized that timing is key; if sufficient aPC is allowed to interact with fVa and/or fVIIIa prior to their involvement in prothrombinase/tenase complex activity, its anticoagulant function may be enhanced as these complexes provide some protection from aPC proteolysis [58]–[60]. What bearing this has *in vivo* in either on healthy or trauma-altered hemostasis remains unclear.

The results presented here should be interpreted with caution given the limitations inherent in characterizing complex and dynamic physiology with *ex vivo* coagulation tests. Nevertheless, the modest increase in aPC levels observed in patients with ATC is unlikely to be the main cause of this coagulopathy. Our results are in line with those found by Gruber et al. which demonstrated in baboon studies that high plasma levels (8.6 nM) following exogenously delivered aPC decreased thrombosis (measured via APTT and fibrin deposition) and platelet aggregation [48]. However, studies by Gruber et al. further showed no hemostatic alteration (measured by bleeding times) and therefore suggest concentrations of aPC investigated in our experiments likely fall short of hemostatic perturbation values *in vivo*. Furthermore, the characterization of ATC as a “hypocoagulable” state induced by aPC obscures the fact that activation of PC requires prior robust thrombin generation [11], [61]. ATC is characterized by exaggerated activation of thrombin and eventual depletion over time of multiple coagulation factors, platelets and inhibitors through transition to Coagulopathy of Trauma [3]. It occurs in the setting of shock where loss of hydrostatic pressure alters the net Starling fluid flux which drives progressive autodilution of blood [62]–[64]. Detection of increased aPC may reflect the final stages of a robust physiologic response to a massive hemostatic challenge rather than a primary anticoagulant response. Regardless, our results indicate that platelets provide adequate fV to overcome the effects of very high aPC concentrations, even in the absence of plasma fV. This finding may explain part of the apparent benefit to early platelet transfusion in trauma patients [65], [66]. However, these present results also indicate that plasma fV is significantly resistant to aPC degradation even at pharmacological levels which indicates that the cause of ATC is more complicated than simply exuberant activation of protein C. Taken together, these data illustrate that the function of aPC is to delay clotting by dampening thrombin generation in a non-linear, protein S-dependent fashion [67], which in a healthy vasculature must be sufficient to mitigate thrombotic tendencies. aPC is not a true anticoagulant in the sense that heparin or directed thrombin inhibitors are; even in a closed system, it does not prevent or weaken clotting except at extremely high concentrations.

Critically, therapeutic approaches designed to prevent aPC generation or inhibit aPC efficacy may be counter-productive; the anti-inflammatory and anti-apoptotic cytoprotective effects [68], [69] of aPC may constitute an adaptive response to tissue injury. Additionally, protein C inhibitor (PCI) and α_1_ anti-trypsin (α_1_AT) which readily bind and inhibit aPC activity [70] have not been evaluated for changes in trauma patient blood samples and may play a significant role in mediating elevated aPC effects.

## References

[1] Gando S, Wada H, Thachil J (2013) Differentiating disseminated intravascular coagulation (DIC) with the fibrinolytic phenotype from coagulopathy of trauma and acute coagulopathy of trauma-shock (COT/ACOTS). J Thromb Haemost 11: 826–835. 23522358 10.1111/jth.12190

[2] Bouillon B, Brohi K, Hess JR, Holcomb JB, Parr MJ, et al (2010) Educational Initiative on Critical Bleeding in Trauma: Chicago, July 11–13, 2008. J Trauma 68: 225–230. 20065778 10.1097/TA.0b013e3181c42815

[3] Brohi K, Cohen MJ, Davenport RA (2007) Acute coagulopathy of trauma: mechanism, identification and effect. Curr Opin Crit Care 13: 680–685. 17975390 10.1097/MCC.0b013e3282f1e78f

[4] Hess JR, Brohi K, Dutton RP, Hauser CJ, Holcomb JB, et al (2008) The coagulopathy of trauma: a review of mechanisms. J Trauma 65: 748–754. 18849786 10.1097/TA.0b013e3181877a9c

[5] Pidcoke HF, Aden JK, Mora AG, Borgman MA, Spinella PC, et al (2012) Ten-year analysis of transfusion in Operation Iraqi Freedom and Operation Enduring Freedom: increased plasma and platelet use correlates with improved survival. J Trauma Acute Care Surg 73: S445–452. 23192068 10.1097/TA.0b013e3182754796

[6] Cohen MJ, Bir N, Rahn P, Dotson R, Brohi K, et al (2009) Protein C depletion early after trauma increases the risk of ventilator-associated pneumonia. J Trauma 67: 1176–1181. 20009664 10.1097/TA.0b013e3181c1c1bc

[7] Brohi K, Singh J, Heron M, Coats T (2003) Acute traumatic coagulopathy. J Trauma 54: 1127–1130. 12813333 10.1097/01.TA.0000069184.82147.06

[8] Brohi K, Cohen MJ, Ganter MT, Schultz MJ, Levi M, et al (2008) Acute coagulopathy of trauma: Hypoperfusion induces systemic anticoagulation and hyperfibrinolysis. J Trauma 64: 1211–1217. 18469643 10.1097/TA.0b013e318169cd3c

[9] Chesebro BB, Rahn P, Carles M, Esmon CT, Xu J, et al (2009) Increase in activated protein C mediates acute traumatic coagulopathy in mice. Shock 32: 659–665. 19333141 10.1097/SHK.0b013e3181a5a632 PMC3574570

[10] Cohen MJ, Call M, Nelson M, Calfee CS, Esmon CT, et al (2012) Critical role of activated protein C in early coagulopathy and later organ failure, infection and death in trauma patients. Ann Surg 255: 379–385. 22133894 10.1097/SLA.0b013e318235d9e6 PMC3549308

[11] Stearns-Kurosawa DJ, Kurosawa S, Mollica JS, Ferrell GL, Esmon CT (1996) The endothelial cell protein C receptor augments protein C activation by the thrombin-thrombomodulin complex. Proc Natl Acad Sci U S A 93: 10212–10216. 8816778 10.1073/pnas.93.19.10212 PMC38363

[12] van 't Veer C, Golden NJ, Kalafatis M, Mann KG (1997) Inhibitory mechanism of the protein C pathway on tissue factor-induced thrombin generation. Synergistic effect in combination with tissue factor pathway inhibitor. J Biol Chem 272: 7983–7994. 9065469 10.1074/jbc.272.12.7983

[13] Hockin MF, Kalafatis M, Shatos M, Mann KG (1997) Protein C activation and factor Va inactivation on human umbilical vein endothelial cells. Arterioscler Thromb Vasc Biol 17: 2765–2775. 9409254 10.1161/01.atv.17.11.2765

[14] Campbell JE, Brummel-Ziedins KE, Butenas S, Mann KG (2010) Cellular regulation of blood coagulation: a model for venous stasis. Blood 116: 6082–6091. 20864579 10.1182/blood-2010-01-266395 PMC3031393

[15] Panteleev MA, Ovanesov MV, Kireev DA, Shibeko AM, Sinauridze EI, et al (2006) Spatial propagation and localization of blood coagulation are regulated by intrinsic and protein C pathways, respectively. Biophys J 90: 1489–1500. 16326897 10.1529/biophysj.105.069062 PMC1367302

[16] Taylor FB Jr, Lockhart MS (1985) Whole blood clot lysis: in vitro modulation by activated protein C. Thromb Res. 37: 639–649. 10.1016/0049-3848(85)90193-8 3838829

[17] Taylor FB Jr, Lockhart MS (1985) A new function for activated protein C: activated protein C prevents inhibition of plasminogen activators by releasate from mononuclear leukocytes—platelet suspensions stimulated by phorbol diester. Thromb Res 37: 155–164. 3920776 10.1016/0049-3848(85)90042-8

[18] Brohi K, Cohen MJ, Ganter MT, Matthay MA, Mackersie RC, et al (2007) Acute traumatic coagulopathy: initiated by hypoperfusion: modulated through the protein C pathway? Ann Surg 245: 812–818. 17457176 10.1097/01.sla.0000256862.79374.31 PMC1877079

[19] Bajzar L, Nesheim ME, Tracy PB (1996) The profibrinolytic effect of activated protein C in clots formed from plasma is TAFI-dependent. Blood 88: 2093–2100. 8822928

[20] Mosnier LO (2011) Platelet Factor 4 Inhibits Thrombomodulin-dependent Activation of Thrombin-activatable Fibrinolysis Inhibitor (TAFI) by Thrombin. Journal of Biological Chemistry 286: 502–510. 21041299 10.1074/jbc.M110.147959 PMC3013010

[21] Urano T, Castellino FJ, Ihara H, Suzuki Y, Ohta M, et al (2003) Activated protein C attenuates coagulation-associated over-expression of fibrinolytic activity by suppressing the thrombin-dependent inactivation of PAI-1. J Thromb Haemost 1: 2615–2620. 14675098 10.1046/j.1538-7836.2003.00443.x

[22] Griffin JH, Fernandez JA, Gale AJ, Mosnier LO (2007) Activated protein C. J Thromb Haemost. 5 Suppl 173–80. 10.1111/j.1538-7836.2007.02491.x 17635713

[23] Macias WL, Dhainaut JF, Yan SC, Helterbrand JD, Seger M, et al (2002) Pharmacokinetic-pharmacodynamic analysis of drotrecogin alfa (activated) in patients with severe sepsis. Clin Pharmacol Ther 72: 391–402. 12386641 10.1067/mcp.2002.128148

[24] Dhainaut JF, Yan SB, Margolis BD, Lorente JA, Russell JA, et al (2003) Drotrecogin alfa (activated) (recombinant human activated protein C) reduces host coagulopathy response in patients with severe sepsis. Thromb Haemost 90: 642–653. 14515185 10.1160/TH02-11-0270

[25] Cornet AD, Hofstra JJ, Vlaar AP, Tuinman PR, Levi M, et al (2013) Activated protein C attenuates pulmonary coagulopathy in patients with acute respiratory distress syndrome. J Thromb Haemost 11: 894–901. 23433188 10.1111/jth.12179 PMC9906436

[26] Camire RM, Kalafatis M, Cushman M, Tracy RP, Mann KG, et al (1995) The mechanism of inactivation of human platelet factor Va from normal and activated protein C-resistant individuals. J Biol Chem 270: 20794–20800. 7657663 10.1074/jbc.270.35.20794

[27] Camire RM, Kalafatis M, Simioni P, Girolami A, Tracy PB (1998) Platelet-derived factor Va/Va Leiden cofactor activities are sustained on the surface of activated platelets despite the presence of activated protein C. Blood. 91: 2818–2829. 9531592

[28] Gould WR, Silveira JR, Tracy PB (2004) Unique in vivo modifications of coagulation factor V produce a physically and functionally distinct platelet-derived cofactor: characterization of purified platelet-derived factor V/Va. J Biol Chem 279: 2383–2393. 14594814 10.1074/jbc.M308600200

[29] Duckers C, Simioni P, Spiezia L, Radu C, Dabrilli P, et al (2010) Residual platelet factor V ensures thrombin generation in patients with severe congenital factor V deficiency and mild bleeding symptoms. Blood 115: 879–886. 19861681 10.1182/blood-2009-08-237719

[30] Zivelin A, Gitel S, Griffin JH, Xu X, Fernandez JA, et al (1999) Extensive venous and arterial thrombosis associated with an inhibitor to activated protein C. Blood. 94: 895–901. 10419879

[31] Cushman M, Costantino JP, Bovill EG, Wickerham DL, Buckley L, et al (2003) Effect of tamoxifen on venous thrombosis risk factors in women without cancer: the Breast Cancer Prevention Trial. Br J Haematol 120: 109–116. 12492585 10.1046/j.1365-2141.2003.03976.x

[32] Schuepbach RA, Feistritzer C, Brass LF, Riewald M (2008) Activated protein C-cleaved protease activated receptor-1 is retained on the endothelial cell surface even in the presence of thrombin. Blood 111: 2667–2673. 18089851 10.1182/blood-2007-09-113076 PMC2254534

[33] Gale AJ, Cramer TJ, Rozenshteyn D, Cruz JR (2008) Detailed mechanisms of the inactivation of factor VIIIa by activated protein C in the presence of its cofactors, protein S and factor V. J Biol Chem. 283: 16355–16362. 10.1074/jbc.M708985200 PMC2423237 18424440

[34] Cramer TJ, Griffin JH, Gale AJ (2010) Factor V is an anticoagulant cofactor for activated protein C during inactivation of factor Va. Pathophysiol Haemost Thromb 37: 17–23. 20501981 10.1159/000315141 PMC2974842

[35] Mustard JF, Perry DW, Ardlie NG, Packham MA (1972) Preparation of suspensions of washed platelets from humans. Br J Haematol 22: 193–204. 4333433 10.1111/j.1365-2141.1972.tb08800.x

[36] Wolberg AS, Gabriel DA, Hoffman M (2002) Analyzing fibrin clot structure using a microplate reader. Blood Coagul Fibrinolysis 13: 533–539. 12192305 10.1097/00001721-200209000-00008

[37] Tracy PB, Eide LL, Bowie EJ, Mann KG (1982) Radioimmunoassay of factor V in human plasma and platelets. Blood 60: 59–63. 7082847

[38] Foley JH, Butenas S, Mann KG, Brummel-Ziedins KE (2012) Measuring the mechanical properties of blood clots formed via the tissue factor pathway of coagulation. Anal Biochem 422: 46–51. 22266209 10.1016/j.ab.2011.12.036 PMC3778664

[39] Butenas S, Branda RF, van't Veer C, Cawthern KM, Mann KG (2001) Platelets and phospholipids in tissue factor-initiated thrombin generation. Thromb Haemost 86: 660–667. 11522019

[40] Kurosawa S, Stearns-Kurosawa DJ, Carson CW, D'Angelo A, Della Valle P, et al (1998) Plasma levels of endothelial cell protein C receptor are elevated in patients with sepsis and systemic lupus erythematosus: lack of correlation with thrombomodulin suggests involvement of different pathological processes. Blood 91: 725–727. 9427734

[41] Liaw PC, Neuenschwander PF, Smirnov MD, Esmon CT (2000) Mechanisms by which soluble endothelial cell protein C receptor modulates protein C and activated protein C function. J Biol Chem 275: 5447–5452. 10681521 10.1074/jbc.275.8.5447

[42] Lenting PJ, van Mourik JA, Mertens K (1998) The life cycle of coagulation factor VIII in view of its structure and function. Blood 92: 3983–3996. 9834200

[43] Lenting PJ, CJ VANS, Denis CV (2007) Clearance mechanisms of von Willebrand factor and factor VIII. J Thromb Haemost 5: 1353–1360. 17425686 10.1111/j.1538-7836.2007.02572.x

[44] Rosing J, Tans G, Govers-Riemslag JW, Zwaal RF, Hemker HC (1980) The role of phospholipids and factor Va in the prothrombinase complex. J Biol Chem 255: 274–283. 7350159

[45] Cui J, O'Shea KS, Purkayastha A, Saunders TL, Ginsburg D (1996) Fatal haemorrhage and incomplete block to embryogenesis in mice lacking coagulation factor V. Nature. 384: 66–68. 10.1038/384066a0 8900278

[46] Duckers C, Simioni P, Spiezia L, Radu C, Gavasso S, et al (2008) Low plasma levels of tissue factor pathway inhibitor in patients with congenital factor V deficiency. Blood 112: 3615–3623. 18695002 10.1182/blood-2008-06-162453 PMC2572790

[47] Gruber A, Griffin JH, Harker LA, Hanson SR (1989) Inhibition of platelet-dependent thrombus formation by human activated protein C in a primate model. Blood 73: 639–642. 2917194

[48] Gruber A, Harker LA, Hanson SR, Kelly AB, Griffin JH (1991) Antithrombotic effects of combining activated protein C and urokinase in nonhuman primates. Circulation 84: 2454–2462. 1835678 10.1161/01.cir.84.6.2454

[49] D'Angelo A, Lockhart MS, D'Angelo SV, Taylor FB Jr (1987) Protein S is a cofactor for activated protein C neutralization of an inhibitor of plasminogen activation released from platelets. Blood 69: 231–237. 2947643

[50] Komissarov AA, Andreasen PA, Declerck PJ, Kamikubo Y, Zhou A, et al (2008) Redirection of the reaction between activated protein C and a serpin to the substrate pathway. Thromb Res 122: 397–404. 18045665 10.1016/j.thromres.2007.10.012

[51] Berckmans RJ, Nieuwland R, Boing AN, Romijn FP, Hack CE, et al (2001) Cell-derived microparticles circulate in healthy humans and support low grade thrombin generation. Thromb Haemost 85: 639–646. 11341498

[52] Perez-Casal M, Downey C, Fukudome K, Marx G, Toh CH (2005) Activated protein C induces the release of microparticle-associated endothelial protein C receptor. Blood 105: 1515–1522. 15486064 10.1182/blood-2004-05-1896

[53] Perez-Casal M, Thompson V, Downey C, Welters I, Wyncoll D, et al (2011) The clinical and functional relevance of microparticles induced by activated protein C treatment in sepsis. Crit Care 15: R195. 21834973 10.1186/cc10356 PMC3387637

[54] Solymoss S, Nguyen KT (1993) Plasma factor V activation is prevented by activated protein C in the presence of phospholipid vesicles, not platelets. Thromb Haemost 69: 124–129. 8456424

[55] Lozano M, Escolar G, Schwarz HP, Hernandez R, Bozzo J, et al (1996) Activated protein C inhibits thrombus formation in a system with flowing blood. Br J Haematol 95: 179–183. 8857957 10.1046/j.1365-2141.1996.d01-1870.x

[56] Schuerholz T, Friedrich L, Marx G, Kornau I, Sumpelmann R, et al (2007) Effect of Drotrecogin alfa (activated) on platelet receptor expression in vitro. Platelets 18: 373–378. 17654307 10.1080/09537100601100788

[57] Kaiser B, Jeske W, Hoppensteadt DH, Walenga JM, Drohan W, et al (1997) In vitro studies on the effect of activated protein C on platelet activation and thrombin generation. Thromb Res 87: 197–204. 9259110 10.1016/s0049-3848(97)00119-9

[58] Koestenberger M, Gallistl S, Muntean W, Leschnik B, Fritsch P, et al (2005) Additive effects of anticoagulants: recombinant human activated protein C and heparin or melagatran, in tissue factor-activated umbilical-cord plasma. Thromb Haemost 94: 69–74. 16113786 10.1160/TH05-01-0041

[59] Kim PY, Nesheim ME (2010) Down regulation of prothrombinase by activated protein C during prothrombin activation. Thromb Haemost 104: 61–70. 20390226 10.1160/TH09-09-0650 PMC3152479

[60] Bravo MC, Orfeo T, Mann KG, Everse SJ (2012) Modeling of human factor Va inactivation by activated protein C. BMC Syst Biol. 6: 45. 10.1186/1752-0509-6-45 PMC3403913 22607732

[61] Taylor FB Jr, Peer GT, Lockhart MS, Ferrell G, Esmon CT (2001) Endothelial cell protein C receptor plays an important role in protein C activation in vivo. Blood 97: 1685–1688. 11238108 10.1182/blood.v97.6.1685

[62] Carey JS (1973) Physiological hemodilution: interrelationships between hemodynamics and blood volume after acute blood loss. Ann Surg 178: 87–94. 4577818 10.1097/00000658-197307000-00018 PMC1355870

[63] Prist R, Rocha-e-Silva M, Scalabrini A, Coelho IJ, Franca ES, et al (1994) A quantitative analysis of transcapillary refill in severe hemorrhagic hypotension in dogs. Shock 1: 188–195. 7735950 10.1097/00024382-199403000-00006

[64] Isbister JP (1997) Physiology and pathophysiology of blood volume regulation. Transfus Sci 18: 409–423. 10175155 10.1016/S0955-3886(97)00040-4

[65] Cap AP, Spinella PC, Borgman MA, Blackbourne LH, Perkins JG (2012) Timing and location of blood product transfusion and outcomes in massively transfused combat casualties. J Trauma Acute Care Surg 73: S89–94. 22847102 10.1097/TA.0b013e318260625a

[66] Holcomb JB, Wade CE, Michalek JE, Chisholm GB, Zarzabal LA, et al (2008) Increased plasma and platelet to red blood cell ratios improves outcome in 466 massively transfused civilian trauma patients. Ann Surg 248: 447–458. 18791365 10.1097/SLA.0b013e318185a9ad

[67] Mosnier LO, Zampolli A, Kerschen EJ, Schuepbach RA, Banerjee Y, et al (2009) Hyperantithrombotic, noncytoprotective Glu149Ala-activated protein C mutant. Blood 113: 5970–5978. 19244160 10.1182/blood-2008-10-183327 PMC2700330

[68] Joyce DE, Gelbert L, Ciaccia A, DeHoff B, Grinnell BW (2001) Gene expression profile of antithrombotic protein c defines new mechanisms modulating inflammation and apoptosis. J Biol Chem 276: 11199–11203. 11278252 10.1074/jbc.C100017200

[69] Mosnier LO, Zlokovic BV, Griffin JH (2007) The cytoprotective protein C pathway. Blood 109: 3161–3172. 17110453 10.1182/blood-2006-09-003004

[70] Espana F, Gruber A, Heeb MJ, Hanson SR, Harker LA, et al (1991) In vivo and in vitro complexes of activated protein C with two inhibitors in baboons. Blood 77: 1754–1760. 1849759

